# Sphingomyelin and Medullary Sponge Kidney Disease: A Biological Link Identified by Omics Approach

**DOI:** 10.3389/fmed.2021.671798

**Published:** 2021-05-26

**Authors:** Simona Granata, Maurizio Bruschi, Michela Deiana, Andrea Petretto, Gianmarco Lombardi, Alberto Verlato, Rossella Elia, Giovanni Candiano, Giovanni Malerba, Giovanni Gambaro, Gianluigi Zaza

**Affiliations:** ^1^Renal Unit, Department of Medicine, University-Hospital of Verona, Verona, Italy; ^2^Laboratory of Molecular Nephrology, Istituto Pediatrico di Ricovero e Cura a Carattere Scientifico (IRCCS) Istituto Giannina Gaslini, Genova, Italy; ^3^Section of Biology and Genetics, Department of Neuroscience, Biomedicine and Movement Sciences, University of Verona, Verona, Italy; ^4^Core Facilities - Clinical Proteomics and Metabolomics, Istituto Pediatrico di Ricovero e Cura a Carattere Scientifico (IRCCS) Istituto Giannina Gaslini, Genoa, Italy

**Keywords:** medullary sponge kidney, idiopathic calcium nephrolithiasis, metabolomics, sphingomyelin, proteomics

## Abstract

**Background:** Molecular biology has recently added new insights into the comprehension of the physiopathology of the medullary sponge kidney disease (MSK), a rare kidney malformation featuring nephrocalcinosis and recurrent renal stones. Pathogenesis and metabolic alterations associated to this disorder have been only partially elucidated.

**Methods:** Plasma and urine samples were collected from 15 MSK patients and 15 controls affected by idiopathic calcium nephrolithiasis (ICN). Plasma metabolomic profile of 7 MSK and 8 ICN patients was performed by liquid chromatography combined with electrospray ionization tandem mass spectrometry (UHPLC–ESI-MS/MS). Subsequently, we reinterrogated proteomic raw data previously obtained from urinary microvesicles of MSK and ICN focusing on proteins associated with sphingomyelin metabolism. Omics results were validated by ELISA in the entire patients' cohort.

**Results:** Thirteen metabolites were able to discriminate MSK from ICN (7 increased and 6 decreased in MSK vs. ICN). Sphingomyelin reached the top level of discrimination between the two study groups (FC: −1.8, *p* < 0.001). Ectonucleotide pyrophophatase phosphodiesterase 6 (ENPP6) and osteopontin (SPP1) resulted the most significant deregulated urinary proteins in MSK vs. ICN (*p* < 0.001). ENPP6 resulted up-regulated also in plasma of MSK by ELISA.

**Conclusion:** Our data revealed a specific high-throughput metabolomics signature of MSK and indicated a pivotal biological role of sphingomyelin in this disease.

## Introduction

Medullary sponge kidney disease (MSK) is a kidney malformation with a rare frequency in the general population but relatively common in renal stone formers (1). This clinical condition is associated with nephrocalcinosis and renal stones, urinary acidification and concentration alterations, and cysts formation in the precalyceal ducts (1). Its pathogenesis is complex and not fully understood.

MSK development in childhood and its relationship with developmental disorders (e.g., congenital hemihypertrophy and Beckwith–Wiedemann syndrome) and with kidney anomalies (e.g., horse-shoe kidney, unilateral renal aplasia, contralateral congenital small kidney) (2–4) support the hypothesis of an inherited condition. Genetic studies revealed that familial clustering of MSK is common and has an autosomal dominant inheritance with a reduced penetrance and variable expressivity (5, 6). Additionally, mutations in the glial cell line-derived neurotrophic factor (GDNF) and receptor tyrosine kinase (RET) genes, disrupting the “ureteric bud–metanephric mesenchyme” interface, could be responsible of the disease pathogenesis (7, 8).

Moreover, as demonstrated by urinary proteomic analysis, some regulators of epithelial cell differentiation, kidney development, cell migration/adhesion, extracellular matrix organization, and complement may be deregulated in MSK and could serve as non-invasive MSK diagnostic biomarkers (9, 10). Among them laminin subunit alpha 2 (LAMA-2) seems a good candidate.

Laminin, a family of at least 15 αβγ heterotrimeric proteins of the extracellular matrix and a major constituent of the basement membrane (11), mediates the attachment, migration, and organization of cells into tissues during embryonic development by interacting with other extracellular matrix components. Additionally, laminin may have a role in the cysts' formation being responsible of cellular apical pole orientation (12–14). LAMA-2 and other selected proteins could be proposed as diagnostic biomarkers to replace invasive procedures or imaging techniques with low sensitivity (e.g., Intravenous Urography and CT urography).

A subsequent analysis of the protein content of microvesicles/exosomes isolated from urine of MSK and idiopathic calcium nephrolithiasis (ICN) patients identified a core panel of 20 proteins that distinguished the two study groups (15). Among them, three exosome proteins involved in the lectin complement pathway maximized the discrimination between MSK and ICN: Ficolin 1, Mannan-binding lectin serine protease 2 and Complement component 4-binding protein β. This revealed, for the first time, a possible involvement of the complement pathway in MSK. In particular the downregulation of MASP2 together with the upregulation of C4BPB that inhibits the activation of the complement cascade by preventing the formation of the classical C3 and C5 convertases, may reflect the physiological attempt of the kidneys to mitigate the activation of the lectin complement pathway, also to preserve renal function. Indeed, the hyperactivation of complement may induce glomerular and tubulointerstitial injury (16).

Additionally, our group has recently described extra-renal alterations involving the cardiovascular apparatus, the central nervous system, and bone metabolism (17, 18) in MSK patients suggesting that it may be considered a systemic disease. These patients are more prone to develop hypertrophic cardiomyopathy with adipose metaplasia and mitral valve prolapse and bone mineralization defects.

Therefore, to obtain additional information on the molecular mechanisms underlying MSK and to discover systemic factors of this disease, we used an untargeted approach to compare the plasma metabolomic profile of MSK vs. idiopathic calcium nephrolithiasis (ICN) patients, used as control group, and we reinterrogated proteomic raw data previously obtained from urinary microvesicles of MSK and ICN (15) focusing on proteins associated with sphingomyelin metabolism.

## Materials and Methods

### Patients

A total of 15 adult patients with medullary sponge kidney disease (MSK) and 15 with idiopathic calcium nephrolithiasis (ICN) matched for age, gender, and geographical origin followed up at Renal Unit of University/Hospital of Verona were included in the study. The main demographic and clinical characteristics of the patients have been reported in Table 1.

**Table 1:** Main demographic and clinical characteristics of the patients.

	**MSK**	**ICN**	***p***
	**(*n* = 15)**	**(*n* = 15)**	
Males, *n* (%)	10 (66.7)	8 (53.3)	0.709
Age, years, mean (SD)	55.80 (15.50)	57.33 (14.80)	0.784
Serum creatinine, mg/dL, mean (SD)	0.86 (0.17)	0.82 (0.12)	0.420
Urinary Ca, mg/die, mean (SD)	317 (107)	222 (107)	0.045
Urinary protein, mg/dL, median (IQR)	0.11 (0.06, 0.13)	0.09 (0.00, 0.15)	0.835
Urinary volume, ml/die, mean (SD)	2136 (450)	1635 (542)	0.026

The inclusion criteria for the MSK group were the same as described in our previous study (9). Particularly, patients had both kidneys involved, nephrocalcinosis and/or cysts in at least 2 papillae in each kidney. For MSK, patients had papillary precalyceal ectasias on films obtained at least 10 min after contrast medium injection in the absence of compression maneuvers and signs of obstruction. The X-ray films were reviewed by an independent radiologist to confirm the diagnosis. For ICN patients the inclusion criteria were as follows: calcium stone disease, normal serum creatinine and electrolyte concentrations, and urinary pH ≤ 5.5 measured in spot morning urine (after overnight fasting) to exclude tubular acidosis. Major exclusion criteria for ICN were as follows: the presence of endocrine diseases and cystic kidney disorders, nephrocalcinosis, and obstructive nephropathy.

Plasma and urine samples were obtained from all patients enrolled in the study. Whole-blood samples were collected in EDTA-coated tubes. The tubes were centrifuged (1,800 × g for 10 min), and the plasma was extracted and aliquoted. Second morning urine were collected and centrifuged to eliminate cells and debris. The supernatants were divided into aliquots and stored at −80°C until use.

Laboratory data were electronically registered for all patients enrolled. All subjects gave their informed consent for inclusion before they participated in the study. The study was conducted in accordance with the Declaration of Helsinki, and the protocol was approved by the Local Ethics Committee (AOUI Verona, 1312CESC).

### Sample Preparation and Set-Up for Metabolomics

Plasma samples from 7 MSK patients and 8 controls affected by idiopathic calcium nephrolithiasis (ICN) were used for metabolomics using liquid chromatography combined with electrospray ionization tandem mass spectrometry (UHPLC–ESI-MS/MS).

Sample preparation was performed according to standard protocols (19). MS setup: plasma metabolites were detected using liquid chromatography combined with electrospray ionization tandem mass spectrometry (HPLC–ESI-MS/MS). The analytic system consists of an Accela 1250 pump, Accela autosampler and a LTQ Orbitrap Velos mass spectrometer (Thermo Scientific, USA). The analytes were separated on Kinetex C18 100 mm × 2.1 mm × 1.7 um and mobile phase [solvent A: aqueous solution of acetic acid (pH 2); solvent B: methanol] in gradient elution at a flow rate of 300 ul/min. The HPLC elution program was as follows: 5% B (2 Min), 30% B (linear increase in 1 Min), 30% B (5 Min), 5% B (linear decrease in 1 min), 5% B (3 min). The column temperature was maintained at 25°C. The injection volume was 5 ul. The metabolites were detected both in the positive (ESI +) and in the negative (ESI –) ionization mode (20).

### Processing of Raw Data

Raw mass spectrometry (MS) files were processed using XCMS software version 3.2.7.1. Features were associated with known metabolites, when possible, searching their M/Z and RT values in the Metlin data base. Features presenting a missing value rate >20% were removed. Variables showing a poor variation in their values and outlier values were removed through a filtering based on Inter Quartile Range (IQR). Each feature was normalized by median-normalization and scaled by Auto scaling (mean-centered and divided by the standard deviation of each variable) (21). Statistical analyses were performed with MetaboAnalyst software version 4.0 (22).

### ELISA

The concentration of sphingomyelin in plasma and urine, of the entire cohort of patients was determined by ELISA (Abcam, AB133118 and LifeSpan BioScience, LS-F30127) following the protocols provided by the manufacturer. ELISA kits were also used to measure the content of SPP1 and ENPP6 (Abcam, ab100618 and MyBiosource, MBS9327272).

### Statistical and Bioinformatics Analyses

For ELISA data analysis, U-Mann-Whitney test was used to assess differences in the protein levels of sphingomyelin, SPP1 and ENPP6 between the 2 study groups. Results were expressed as median and IQ range. A value of *P* < 0.05 was considered to be statistically significant.

For metabolomics, differences in feature mean values between paired samples (i.e., pre and post run) were tested by estimating a fold-change value between groups and an associated *p*-value. Dysregulated features (fold change ≥ 1.5 and nominal *p* ≤ 0.05, arbitrarily chosen) were plotted in a Cloud plot, reporting intensities of signals between groups.

For *t*-test analysis *p*-values were adjusted for multiple testing using the Benjamini-Hochberg false discovery rate. Principal component analysis (PCA) was used to explore the data and identify any possible resemblances among subjects, based on the value of all features. Statistical analysis of metabolite-associated features was performed using XCMS and MetaboAnalyst (v.4.0) (22).

Enrichment analysis of the biological process was performed using information of the Kegg database.

For urine proteomic analysis, we have re-interrogated our previous proteomic datasets obtained from whole urinary and urinary microvesicles isolated from 15 MSK and 15 ICN (9, 15).

In particular, mass spectrometry data were analyzed as previously reported (15). Then, the fold change of the identified proteins associated to the sphingomyelin metabolism (23–25) and their −Log10 *P*-values were visualized in a volcano plot. The proteomic profile, after Z-score normalization, of these associated proteins were visualized by Heatmap diagram. Finally, Support Vector Machine (SVM) was used to identify a rank list of sphingomyelin metabolism proteins for discrimination between MSK and ICN samples. Proteins were considered to be significantly differentially expressed between the two conditions with power of 80% and an adjusted *P* ≤ 0.05 in the *T*-test after correction for multiple interactions (Benjamini-Hochberg) and a fold change of ≥2. In addition, the proteins needed to show at least 70% identity in the samples in one of two conditions. In SVM a 4-fold cross-validation approach was applied to estimate the prediction and classification accuracy of ranked list of statistically significant proteins. All proteomic statistical analysis were performed using OriginLab and the latest version of software package R available at the time of the experiments.

## Results

### Metabolomic Profiling Discriminated MSK From ICN Patients

**Figure 1:**
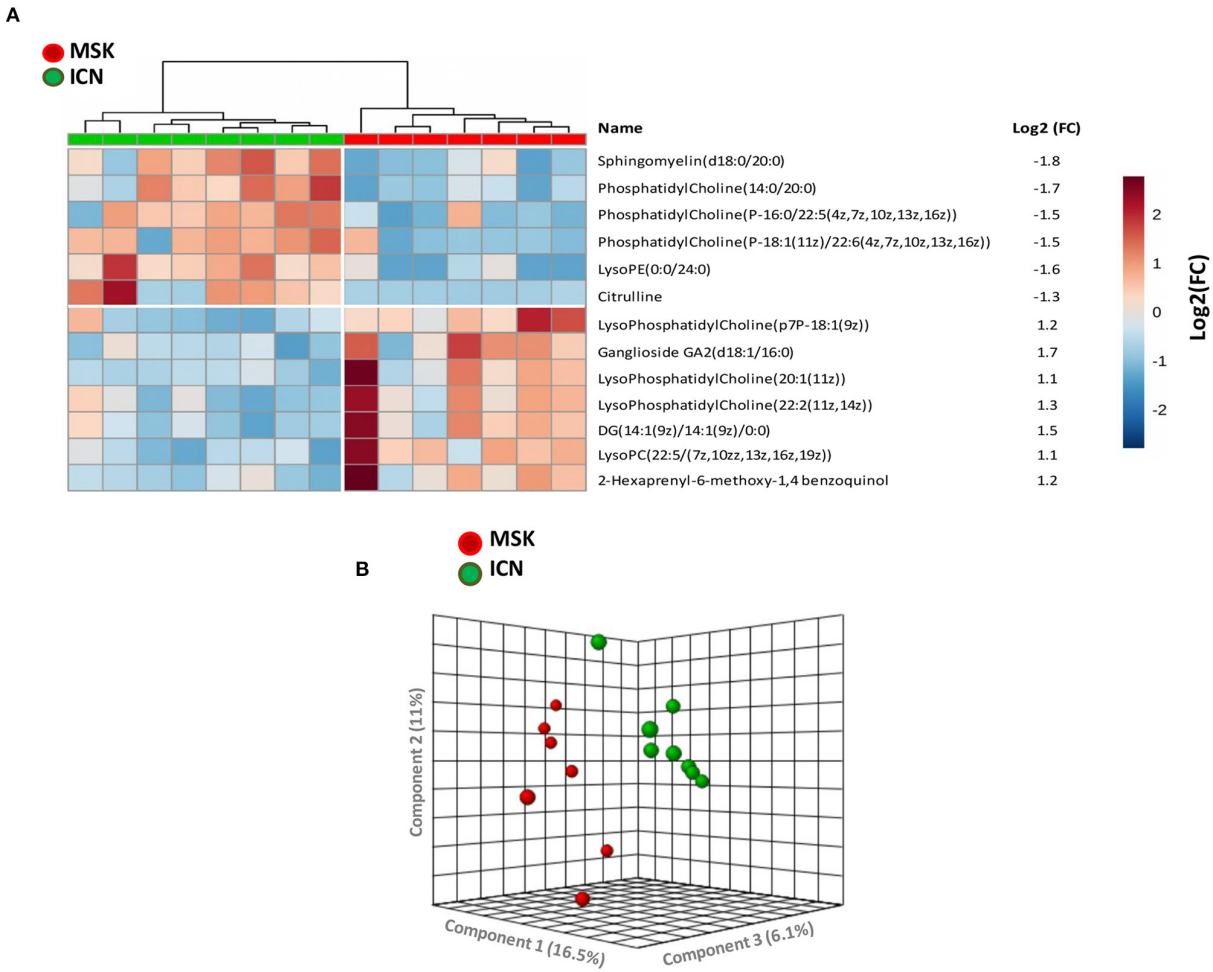
2-D Supervised hierarchical clustering and Principal Component Analysis (PCA) discriminating MSK from ICN patients. **(A)** 2-D Supervised hierarchical clustering able to discriminate patients with Medullary Sponge Kidney (MSK) from controls (ICN, nephrolithiasis). Patients are represented as vertical columns, with red symbols indicating MSK patients (*n* = 7) and green symbols ICN (*n* = 8). Thirteen metabolites (rows) were used for hierarchical grouping. The scale intensity of the metabolites is depicted according to the color key shown on the right. Red indicates high intensity level; blue, low intensity level. The figure also shows the mean levels of the fold change of expression of each metabolite in the MSK group compared to controls. **(B)** PCA plot built using the 13 metabolites selected by statistical analysis showed high discrimination accuracy between the two study groups.

After missing values estimation, 4,005 signals have been obtained and subjected to statistical analysis. Thirteen metabolites were able to clearly discriminate MSK from ICN (7 metabolites increased and 6 decreased in MSK compared to ICN patients) (Figure 1A). Using the principal Component Analysis (PCA) plot of the selected 13 metabolites, it was possible to clearly differentiate MSK from ICN (Figure 1B).

Sphingomyelin, which level was significantly decreased in MSK vs. ICN, reached the higher level of discrimination between the two study groups (Log2 fold change: −1.8, *p* < 0.001).

### ELISA for Sphingomyelin Confirmed Metabolomics Results

To validate metabolomics data, we performed ELISA on plasma samples collected from all enrolled patients (15 MSK and 15 ICN).

**Figure 2:**
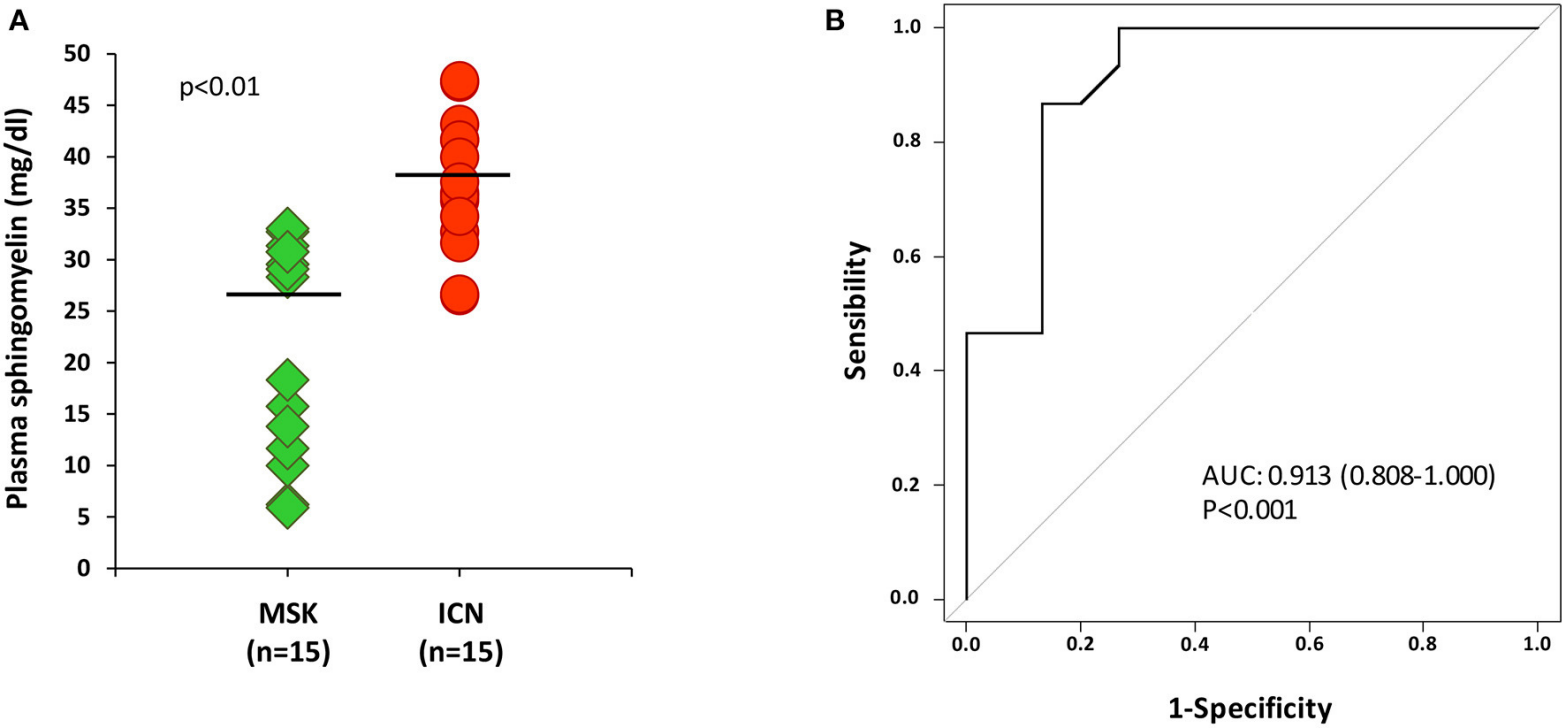
ELISA for sphingomyelin validated metabolomic results. **(A)** Dot plot shows sphingomyelin plasma levels (mg/dl) in 15 patients with MSK (green) and 15 with nephrolithiasis (ICN, red). Solid lines indicate median values. The *p* value was calculated using the *t*-test. **(B)** ROC curve analysis of plasma sphingomyelin revealed that the reported values allow to discriminate MSK from the ICN.

Data analysis clearly demonstrated that the level of sphingomyelin was significantly lower in MSK patients than in ICN [Median/(IQR) MSK vs. ICN, 28.33 (12.73, 30.76) vs. 36.52 (33.48, 41.67), *p* < 0.01] (Figure 2A). ROC curve showed that the level of sphingomyelin can discriminate MSK from ICN patients (Figure 2B). The AUC, 95% CI and *p* values for the ROC analysis were 0.913, 0.808–1.000, and *p* < 0.001, respectively. The cutoff, sensitivity, specificity were 31.5 mg/dl, 87%, 87%, respectively.

To confirm whether sphingomyelin was reduced also in kidney, we decided to measure its urinary level by ELISA in MSK and ICN patients.

**Figure 3:**
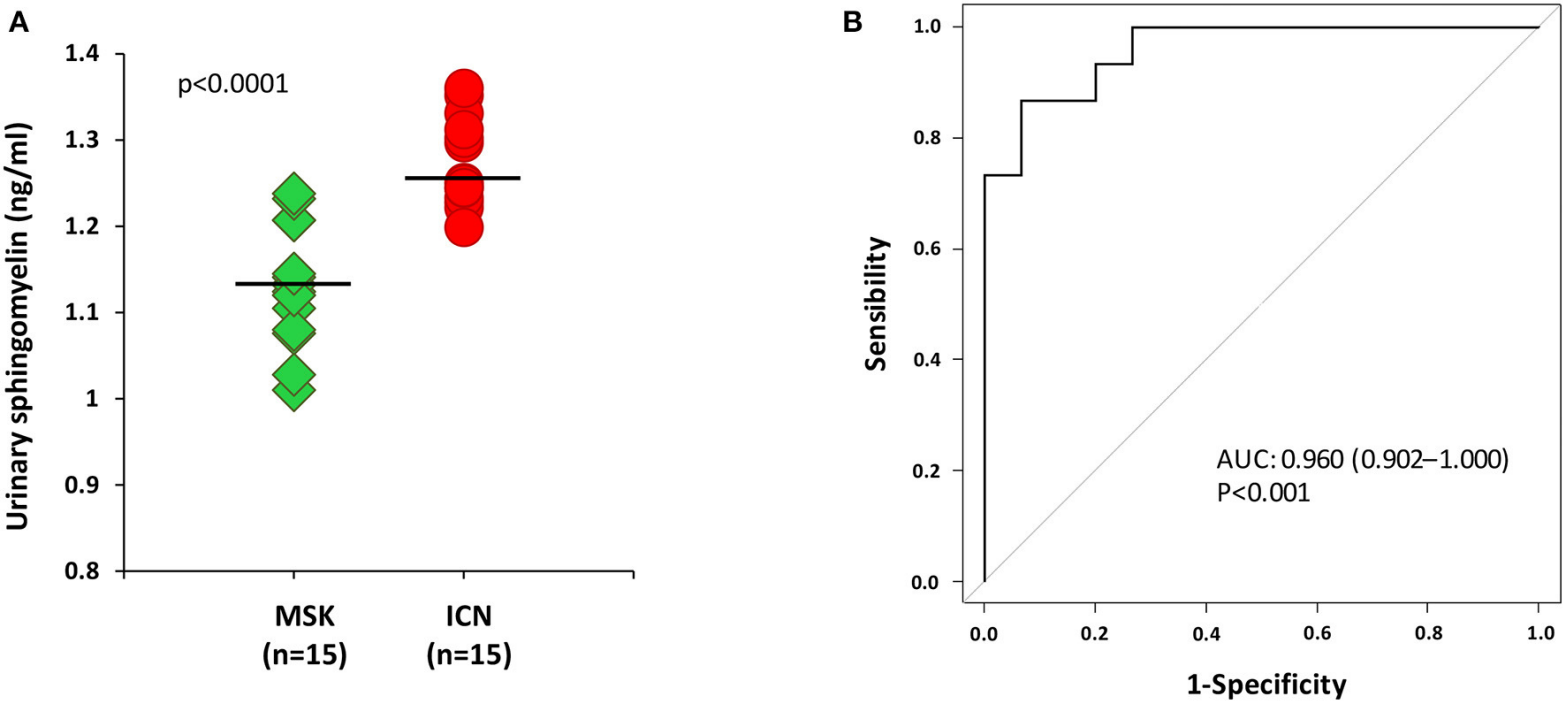
ELISA for the urinary content of sphingomyelin was in accordance with the results obtained in plasma. **(A)** Dot plot shows sphingomyelin urinary levels (ng/ml) measured in 15 patients with MSK and 15 with ICN. Solid lines indicate median values. The *p* value was calculated using the *U* Mann-Whitney test. **(B)** ROC curve analysis of urinary sphingomyelin revealed that the reported values allow to discriminate MSK from the ICN.

According to the results obtained in plasma, the level of sphingomyelin in urine was lower in MSK patients than in ICN (Figure 3A) [Median/(IQR) MSK vs. ICN, 1.13(1.08–1.21) ng/ml vs. 1.25 (1.23–1.31) ng/ml *p* < 0.0001]. The AUC, 95% CI and *p* values in ROC analysis were 0.960, 0.902–1.000, and *p* < 0.001, respectively (Figure 3B). The cutoff, sensitivity, specificity were 1.2 ng/ml, 93%, 87% respectively.

### Proteomic Profile of Urinary Microvesicles Differentiated MSK From ICN

The analysis of urinary proteomic data of MSK and ICN focused on proteins associated with sphingomyelin metabolism did not show any statistically significant difference (Supplementary Methods and Supplementary Figure 1).

Then, since microvesicles seem to be involved in the complex biological machinery associated with MSK (15) and appear highly enriched in sphingomyelin (26), we decided to re-interrogate proteomic raw data previously obtained (15) from urinary microvesicles isolated from MSK and ICN focusing on proteins associated with sphingomyelin metabolism (Supplementary Methods).

**Figure 4:**
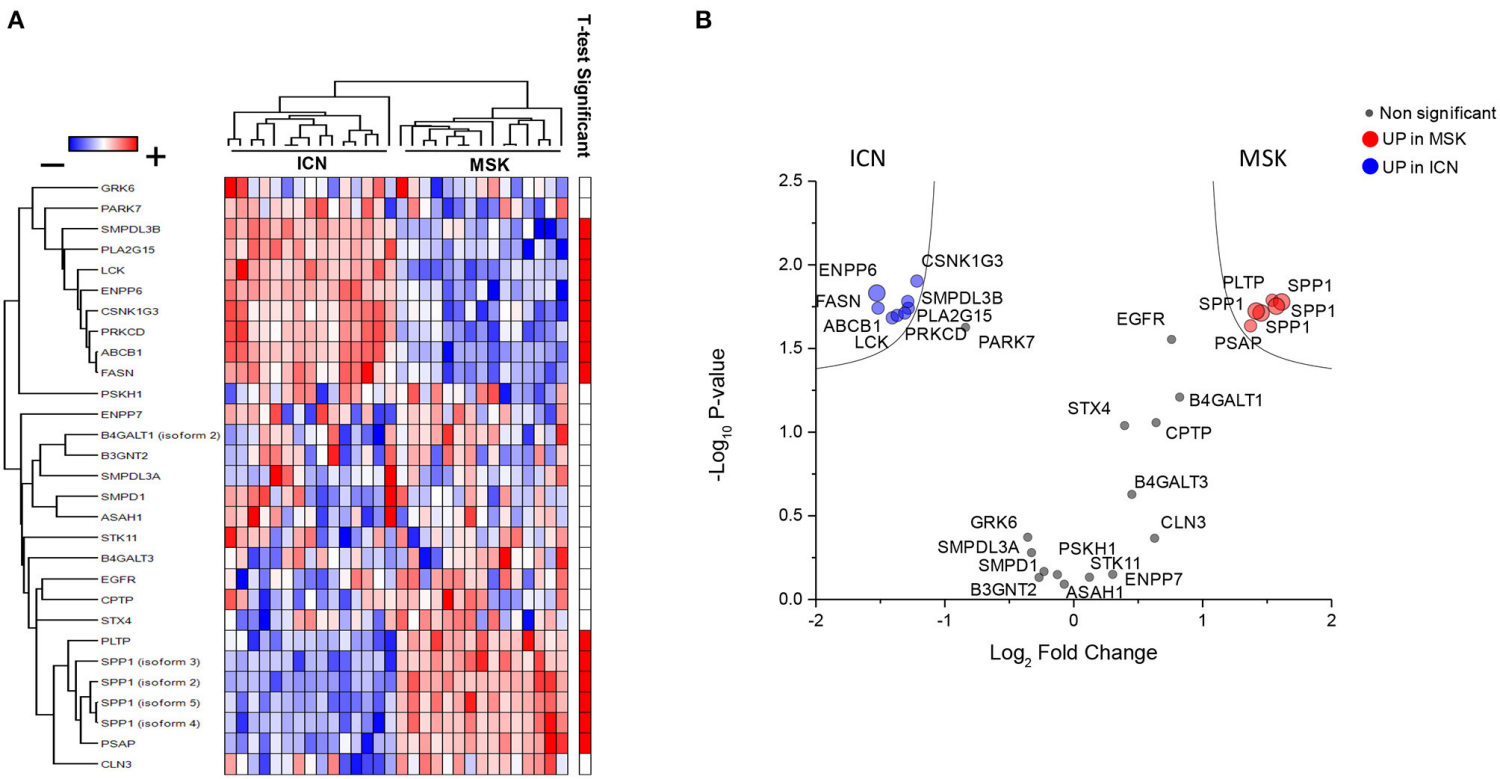
Proteomic profile. **(A)** Heatmap of the 29 proteins associated to sphingomyelin metabolism identified in urinary microvesicles of MSK and ICN patients. Each row represents a protein and each column a sample. Normalized Z-scores of protein abundance are depicted using a pseudocolor scale (red, white, and blue indicating positive equal and negative expression, respectively) compared to each protein value. The dendrogram displays unsupervised hierarchical clustering analysis. Similar sample/proteome-profile values are next to each other. **(B)** Volcano plot of the 29 proteins associated to sphingomyelin metabolism. The plot is based on the relative abundance ratio (log2 fold change) and the *p* value (−log10). Gray, red and blue circles indicate the changes for the non-significant, significant up and down regulated proteins in MSK samples. Black line indicates the limits of statistically significant.

**Figure 5:**
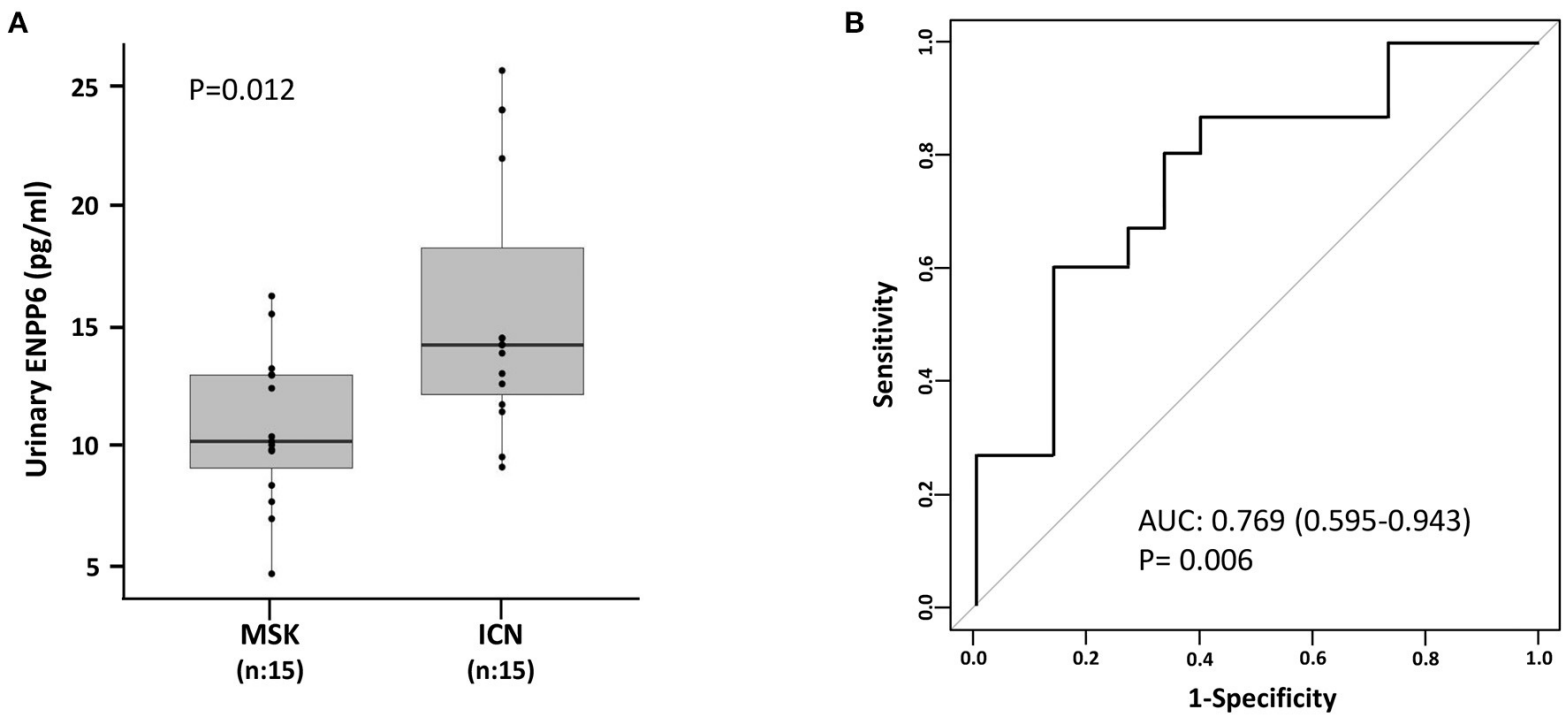
ELISA for the urinary content of ENPP6 confirmed proteomics results. **(A)** Box plot shows ENPP6 urinary levels (pg/ml) measured in 15 patients with MSK and 15 with ICN. Solid lines indicate median values. The *p* value was calculated using the *U* Mann-Whitney test. **(B)** ROC curve analysis of urinary ENPP6 revealed that the reported values allow to discriminate MSK from the ICN.

As shown in Figure 4, twenty-nine proteins associated to sphingomyelin metabolism were identified. Among these, 14 proteins were able to discriminate MSK from ICN patients. SPP1 (Osteopontin) and ENPP6 (Ectonucleotide Pyrophosphatase/Phosphodiesterase 6), two proteins involved in renal morphogenesis, were the top de-regulated proteins identified by the SVM to distinguish between urinary microvesicles isolated from MSK and ICN. In particular, SPP1, in four isoforms (2, 3, 4, and 5) appeared over-expressed, while ENPP6 under-expressed in MSK compared to ICN (Figures 4A,B). ENPP6 down-regulation in MSK was also confirmed by urinary ELISA (Figure 5). Instead, SPP1 levels did not reach statistical differences in MSK vs. ICN (see whole urinary proteomics results in Supplementary Figure 1).

### ELISA Demonstrated Low Levels of ENPP6 in Plasma of MSK vs. ICN Patients

**Figure 6:**
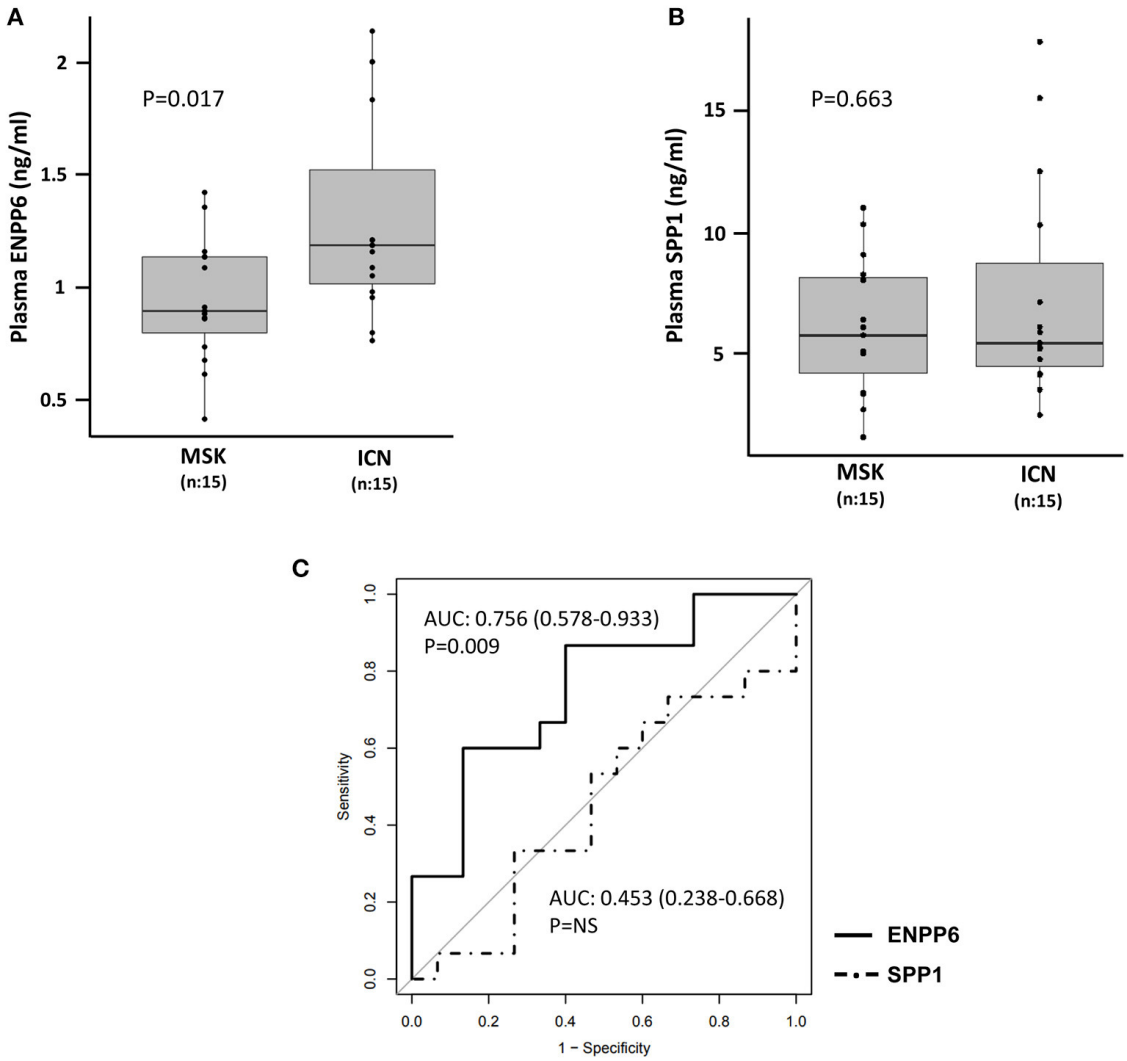
Quantification of ENPP6 and SPP1 by ELISA. Box plots revealed **(A)** higher level of ENPP6 (ng/ml) in ICN vs. MSK and **(B)** no significant difference in the level of SPP1 (ng/ml) in plasma of MSK and ICN. The *p* values were calculated using the *t*-test. **(C)** Analysis of the ROC curve for plasma ENPP6 and SPP1.

Statistical analysis showed that the ENPP6 was under-expressed in plasma of patients with MSK compared to ICN (controls) [Median/(IQR) MSK vs. ICN, 0.9 (0.8, 1.1) vs. 1.2 (1.0, 1.5), *p* = 0.017]. ROC analysis revealed the power of discrimination of this biological factor (Figure 6A). The AUC, 95% CI and *p*-values for the ROC analysis were 0.756, 0.578–0.933, and *p* = 0.009, respectively. The cutoff, sensitivity, specificity were 0.93 ng/ml, 87%, 60% respectively.

Contrarily, no difference was found at plasma level in SPP1 between the two groups [Median/(IQR) MSK vs. ICN, 7.06 (6.18, 10.00) vs. 7.34 (5.93, 9.48), *p* = 0.663] (Figure 6B). The AUC and 95% CI for the ROC analysis were 0.453, 0.238–0.668. The cutoff, sensitivity, specificity were 5.3 ng/ml, 93%, 27% respectively (Figure 6C).

## Discussion

This study demonstrated, for the first time, the involvement of the sphingolipid metabolic pathway in medullary sponge kidney (MSK) disease pathogenesis and offered new insights into the biological machinery associated with this complex and neglected disorder.

Results of our omics research strategy revealed that several metabolites (sphingomyelin, phosphatidylcholine, lysophosphatidylethanolamine, citrulline, lysophosphatidylcholine, Ganglioside GA2, diglyceride, 2-Hexaprenyl-6-methoxy-1,4-benzoquinol) were able to clearly discriminate MSK from ICN. Sphingomyelin, a sphingolipid and major component of mammalian cells, resulted the most down-regulated.

Sphingomyelin can be synthesized from ceramide by sphingomyelin synthase (SMS) types 1 and 2 (27). SMS1 is constitutively expressed in the Golgi apparatus and is involved in the homeostatic synthesis of SM (28), while the expression of SMS2 in the Golgi depends on numerous stimuli (29). The reverse process of sphingomyelin hydrolysis to ceramide and phosphorylcholine is induced by different isoforms of sphingomyelinase and represents an alternative route for the synthesis of ceramide (30).

At our knowledge, this is the first report describing a specific metabolic fingerprint of MSK and the possible involvement of sphingomyelin in the MSK-associated biological impairment. Instead, alterations in the metabolism of sphingomyelin and other sphingolipids have been extensively reported in other diseases including neurodegenerative [Alzheimer's disease (31), Parkinson's (32), multiple sclerosis (33), Lewy body dementia (34)], vascular (35), and bone disorders (30).

Sphingomyelin and ceramide are implicated in the survival of osteoclasts (36) and in the mineralization of the extracellular matrix. As demonstrated in animal models, a neutral Sphingomyelinase 2 deficiency is responsible for defects in bone and dental mineralization, probably due to deficiencies in ceramide and phosphocholine synthesis secondary to the degradation of sphingomyelin (37).

The involvement of sphingomyelin in MSK could partially explain the pathogenesis of systemic alterations observed in these patients. MSK patients, in fact, seem to have an altered neuroprotection capability against oxidative stress/ischemia and several bone metabolic defects (17, 18). As previously reported by our group, some MSK patients may develop central nervous system alterations (17) with the genetic derangement of the RET–GDNF axis having a possible pathogenetic role (38). Sphingomyelin, involved in several neurodegenerative disorders (39), may also contribute to the development of these clinical features.

As previously pointed out, about 75% of patients with MSK have alterations in bone mineralization (60% osteopenia and about 15% osteoporosis) in the absence of common risk factors such as menopause or hyperparathyroidism and also due to the persistent hypercalciuria (18). This high secretion of calcium may be the result of the renal calcium-handling defect (1), absorptive hypercalciuria (40), and defective urinary acidification (41) that characterize these patients.

The reduction of sphingomyelin, observed in MSK patients, could represent an adaptive response of the bone tissue to modulate osteoblastic/osteoclastic activity in the presence of a negative calcium balance and altered tubular acidification typical changes in the kidneys of these patients.

Sphingomyelin and some of its associated proteins, enriched in microvesicles [characterized by a high content of sphingolipids (42)] resulted also deregulated in the urines of our MSK patients demonstrating a possible ubiquitously alteration of this pathway in these patients. The absence of statistical difference in the level of the proteins associated with sphingomyelin metabolism in whole urinary proteomics may be due to the fact that it is mainly synthesized/compartmentalized in Golgi by the biological/biochemical machinery of the renal epithelial tubular cells and then, probably, excreted in urine. Additionally, we cannot exclude that part of the sphingomyelin detected in urine by ELISA could derive from plasma. Further studies are necessary to better understand this issue.

Interestingly, ectonucleotide pyrophophatase phosphodiesterase 6 (ENPP6) and osteopontin (SPP1) resulted the most down- and high-regulated protein, respectively.

ENPP6 belongs to a family of 7 phosphodiesterases involved in multiple cellular processes. ENPP6 hydrolyzes only choline-containing lysophospholipids to phosphocholine and monoacylglyceride (43). It is expressed in multiple tissues including the heart, central nervous system, kidney and bone tissue. In the kidney it is mostly expressed on the luminal side of proximal tubular epithelial cells. It is possible that ENPP6 in the kidney contributes to the reabsorption of choline by hydrolyzing glycerophosphocholine in the primary urine (43).

ENPP6, then, would participate in the synthesis of inorganic phosphate, a constituent of the bone matrix, through the metabolism of phosphocholine (44) and its higher level in ICN could predispose these patients to stone forming (45).

SPP1 is a widely expressed and multifunctional phosphorylated acid glycoprotein, it regulates the synthesis of bone matrix and the activity of osteoclasts. Osteopontin increases bone resorption by stimulating osteoclastogenesis and by anchoring osteoclasts to the matrix (46, 47). SPP1 in physiological conditions is expressed in the distal nephron, especially at the level of the thick segment of the loop of Henle.

In humans, there is an increased expression of osteopontin in the urine in several kidney diseases: hypertensive nephropathy, renal carcinoma, membranous glomerulonephritis, IgA nephropathy, lupus nephritis and in mouse models of ADPKD.

In addition, high expression of osteopontin in urine from MSK has been already reported by our group (48). Then, Ricci et al., have described a similar up-regulation in a group of pediatric patients suffering from ADTKD (Autosomal dominant Tubulointerstitial Kidney Disease) associated with a mutation of HNF1B, a hereditary tubulointerstitial nephritis with cystic dilatation of the renal tubules (49) and in kidney from rat model of ADPKD (50). It is possible that this protein could mediate the cyst formation in MSK patients similarly to ADPKD. The absence of a significant difference in its plasma level in our MSK vs. ICN patients could be explained by a possible paracrine urinary effect of this protein in MSK.

Therefore, our study, although performed on a small cohort of patients (particularly in metabolomics), highlighted a specific metabolic profile associated with MSK and confirmed our hypothesis that this disease could have systemic implications. Sphingomyelin, then, could represent a new disease pathogenetic element and a potential novel biomarker or therapeutic target. Further studies that could employ urinary metabolomics and *in vitro/in vivo* functional experiments, need to be undertaken to validate our research hypothesis and to translate our results into the daily clinical practice.

## Data Availability Statement

The datasets presented in this study can be found in online repositories. The names of the repository/repositories and accession numbers can be found below: Proteomics data are available at PRIDE repository, https://www.ebi.ac.uk/pride/. Accession: PXD025744 and PXD025547. Metabolomics data supporting the conclusions of this article will be made available by the authors, without undue reservation.

## Ethics Statement

The studies involving human participants were reviewed and approved by Comitato Etico per la Sperimentazione Clinica delle Province di Verona e Rovigo Azienda Ospedaliera Universitaria Integrata Verona. The patients/participants provided their written informed consent to participate in this study.

## Author Contributions

SG and GZ: conceptualization and draft of the manuscript. SG, MB, MD, AP, AV, and RE: investigation. SG, MB, MD, GL, GC, and GZ: formal analysis. GM: critical revision of the final version of the manuscript. GZ, GG, and GM: review and editing of the manuscript. GZ: project administration. All authors contributed to the article and approved the submitted version.

## Conflict of Interest

The authors declare that the research was conducted in the absence of any commercial or financial relationships that could be construed as a potential conflict of interest.
