# Supplementary material for: Sphingomyelin and Medullary Sponge Kidney Disease: A Biological Link Identified by Omics Approach
Source: Front Med (Lausanne). 2021 May 26;8:671798. doi: 10.3389/fmed.2021.671798 (PMC8187918; doi:10.3389/fmed.2021.671798)
Supplement: Supplementary file 1 [file Data_Sheet_1.PDF]

Supplementary Material

Supplementary Figure

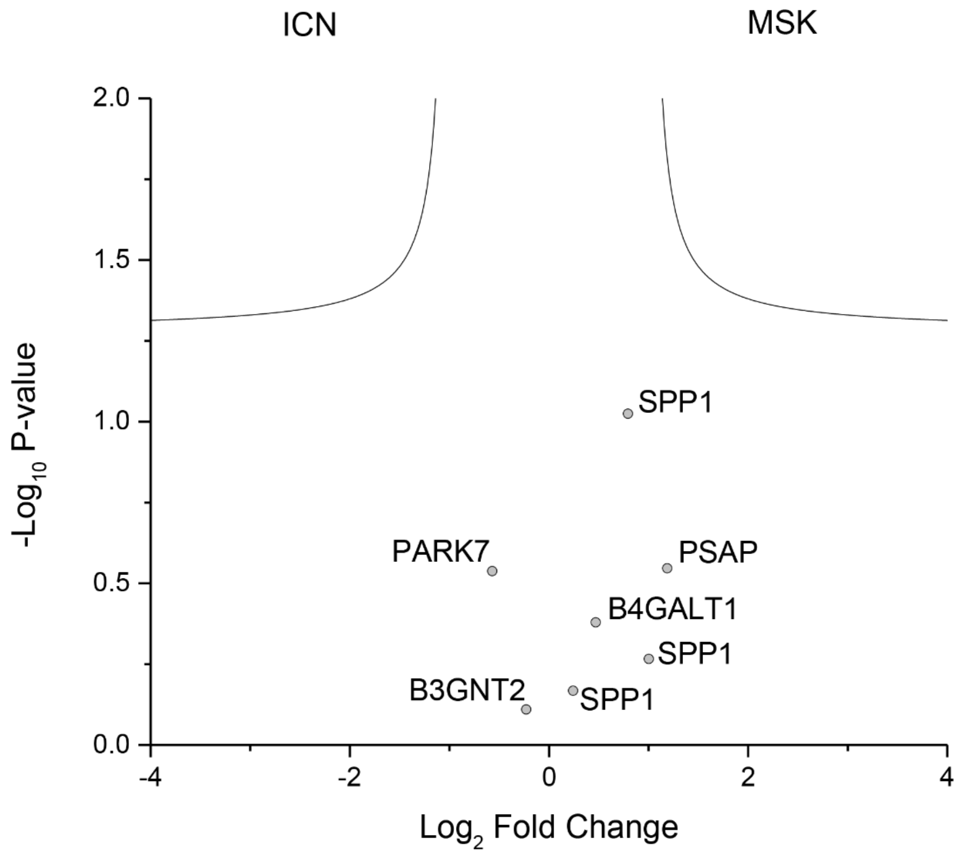

Supplementary Figure 1. The Volcano plot of proteins associated to sphingomyelin metabolism identified in total urine of MSK and ICN patients. Volcano plot of the 5 proteins associated to sphingomyelin metabolism. The plot is based on the fold change (log2) and the P value (–log10). Grey circles indicate the changes for the non-significant proteins in the comparison of the MSK and ICN samples. Black line indicates the limits of statistically significant.
