# Supplementary material for: Sphingomyelin and Medullary Sponge Kidney Disease: A Biological Link Identified by Omics Approach
Source: Front Med (Lausanne). 2021 May 26;8:671798. doi: 10.3389/fmed.2021.671798 (PMC8187918; doi:10.3389/fmed.2021.671798)
Supplement: Supplementary file 2 [file Data_Sheet_2.PDF]

Supplementary Material

Supplementary Methods

Urinary proteomics

Urine collection and fractionation

Second morning urine from MSK disease and ICN patients were collected and centrifuged at 1000g for 10 minutes to eliminate cell debris according to standard protocols of European kidney and urine proteomics (http://www.eurokup.org). The supernatants were divided into several aliquots of 30 ml and stored at -80°C until use [1].

Mass spectrometry

A linear trap Quadrupole Orbitrap Velos Pro mass spectrometer (ThermoFisher Scientific, Waltham, MA) was used for analysis of all samples. Samples were solubilized in 0.1 ml of 4% wt/vol sodium dodecylsulfate, 50 mM dithiothreitol, and 0.1 M Tris/HCl, pH 7.6, at 90°C for 5 minutes and sonicated and processed by the Filter Aided Sample Preparation procedure using 30k Vivacom filtration devices. Briefly, 50-ml aliquots were mixed with 0.2 ml of 8 M urea in 0.1 M Tris/HCl, pH 8.5 (UT solution), loaded into the filtration devices and centrifuged at 14,000g for 15 minutes. Concentrates were diluted in the devices with 0.2 ml of UT solution and centrifuged again. After centrifugation, concentrates were mixed with 0.1 ml of 50 mM iodoacetamide in UT solution and incubated in darkness at room temperature for 30 minutes followed by centrifugation for 15 minutes. Then the concentrates were diluted with 0.2 ml of 8 M urea in 0.1 M Tris/HCl, pH 8.5, and concentrated again. This step was repeated twice. The samples were then diluted with 120 ml of 40 mM NaHCO3 containing 1 mg of trypsin. After an overnight digestion, the peptides were collected by centrifugation of the filter units for 20 minutes, and the filter device was rinsed with 50 ml 0.5 M NaCl and centrifuged to suppress hydrophobic interactions.

Mass spectrometer setup

The linear trap Quadrupole Orbitrap Velos Pro mass spectrometer was operated in the positive ionization mode. Single MS survey scans were performed in the Orbitrap, recording a mass window between 350 and 1650 m/z using a maximal ion injection time of 250 ms. The resolution was set at 60,000, and the automatic gain control was set at 1,000,000 ions. The lock mass option was enabled, allowing the internal recalibration of spectra recorded in the Orbitrap by polydimethyl cyclosiloxane background ions (protonated [Si(CH3)2O)] 6; m/z 445.120025). The experiments were done in data-dependent acquisition mode with alternating MS and MS/MS experiments. The minimum MS signal for triggering MS/MS was set at 500 ions, with the most prominent ion signal selected for MS/MS using an isolation window of 2 Da. The m/z values of signals already selected for MS/MS were put on an exclusion list for 60 seconds using an exclusion window size of ±10 ppm. In all cases, 1 microscan was recorded. Collision-induced dissociation was done with a target value of 5000 ions in the linear ion trap, a maximal ion injection time of 150 ms, normalized collision energy of 35%, a Q-value of 0.25, and an activation time of 10 ms. A maximum of 20 MS/MS experiments was triggered per MS scan.

Proteomic analysis of urinary microvesicles

Isolation of microvesicles

Microvesicles were isolated by centrifugation as previously reported [3]. Briefly, we centrifuged 16 ml aliquots of urine at 16,000×g for 30 min at 16°C in order to remove alive and dead cells, and organelles. The obtained supernatant was centrifuged for 120 min at 22,000×g at 16°C to obtain the microvesicle fraction. The microvesicle-containing pellet was washed in phosphate buffered saline (PBS) and repelleted as above for a total of five wash cycles to obtain a clean microvesicle fraction that were stored at -80°C until use [4].

Mass spectrometry

Samples were processed with in-StageTip (iST) method with two poly (styrene divinylbenzene) reversed-phase sulfonate (SDB-RPS) disks. The microvesicle- and exosome-containing pellets were solubilized with a solution containing 10 mM Tris(2-carboxyethyl)phosphine, 40 mM chloro-acetamide, 100 mM Tris (pH 8.5), 2% sodium deoxycholate. Lysis, reduction, and alkylation of the

samples were performed in a single step and then loaded into the StageTip. The samples were then diluted with a buffer containing 25 mM Tris (pH 8.5) and 1 µg of trypsin. After acidification step with 1% trifluoroacetic acid (TFA) the samples were washed with 0.2% TFA three times. The proteins were eluted in 60 µl 5% v/v ammonium hydroxide containing 80% v/v acetonitrile. The desalted peptides were dried in a speed vacuum and resuspended in 2% acetonitrile containing 0.2% formic acid (FA). They were then separated on a 50-cm reversed-phase Easy Spray column (75-µm internal diameter × 50 cm; 2 µm/100 Å C18) on an Ultimate 3000 RSLCnano device (Thermo Fisher Scientific, Waltham, MA, USA) with a binary buffer system comprising buffer A (0.1% FA) and buffer B (80% acetonitrile, 5% dimethylsulfoxide, 0.1% FA). The program consisted of a 70-min gradient (2–45% buffer B) at a flow rate of 250 µl/min, with the column temperature maintained at 60°C. The chromatography system was coupled to an Orbitrap Fusion Tribrid mass spectrometer (Thermo Fisher Scientific), acquiring data in Charge Ordered Parallel Ion aNalysis (CHOPIN) mode. The precursors were ionized using an EASY-spray source held at +2.2 kV and the iICNet capillary temperature was held at 300°C. Single MS survey scans were performed over the mass window 375–1500 m/z with an AGC target of 250,000, a maximum injection time of 50 ms, and a resolution of 120,000 at 200 m/z. Monoisotopic precursor selection was enabled for peptide isotopic distributions, precursors of z=2-5 were selected for 2 s of cycle time, and dynamic exclusion was set to 25 s with a ±10 ppm window set around the precursor. The following CHOPIN conditions were applied: a) if the precursor charge state is 2, then follow with collision-induced dissociation (CID) and scan in the ion trap with an isolation window of 1.8, CID energy of 35% and a rapid ion trap scan rate; b) if the precursor charge state is 3–5 and precursor intensity >500,000, then follow with higher-energy C-trap dissociation (HCD) and scan in the Orbitrap with an isolation window of 1.8, HCD energy of 28% and a resolution of 15000; c) if the precursor charge state is 3–5 and precursor intensity <500,000, then follow with CID as described for option (a). For all MS2 events, the following options were set: “Injection Ions for All Available Parallelizable Time” with an AGC target value of 4000 and a maximum injection time of 250 ms for CID, or an AGC target value of 10,000 and a maximum injection time of 40 ms for HCD.

The mass spectrometry-based proteomics data are available at ProteomeXchange Consortium via the PRIDE [2] partner repository with the dataset identifier PXD025547 (Reviewer account details: Username: reviewer_pxd025547@ebi.ac.uk, Password: 2MMYsICc)

Supplementary Figure

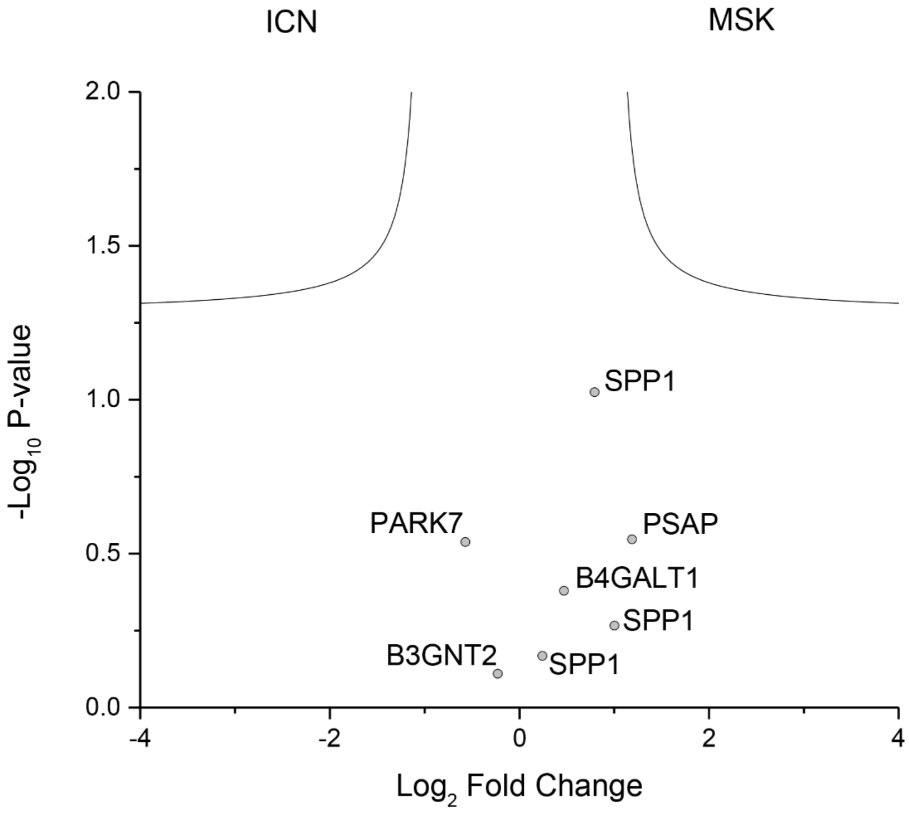

Supplementary Figure 1. The Volcano plot of proteins associated to sphingomyelin metabolism identified in total urine of MSK and ICN patients. Volcano plot of the 5 proteins associated to sphingomyelin metabolism. The plot is based on the fold change (log2) and the P value (–log10). Grey circles indicate the changes for the non-significant proteins in the comparison of the MSK and ICN samples. Black line indicates the limits of statistically significant.

References

1. Fabris A, Bruschi M, Santucci L, Candiano G, Granata S, Dalla Gassa A, Antonucci N, Petretto A, Ghiggeri GM, Gambaro G, Lupo A, Zaza G. Proteomic-based research strategy identified laminin subunit alpha 2 as a potential urinary-specific biomarker for the medullary sponge kidney disease. Kidney Int. 2017 Feb;91(2):459-468

2. Perez-Riverol Y, Csordas A, Bai J, Bernal-Llinares M, Hewapathirana S, Kundu DJ, Inuganti A, Griss J, Mayer G, Eisenacher M, Pérez E, Uszkoreit J, Pfeuffer J, Sachsenberg T, Yilmaz S, Tiwary S, Cox J, Audain E, Walzer M, Jarnuczak AF, Ternent T, Brazma A, Vizcaíno JA. The PRIDE database and related tools and resources in 2019: improving support for quantification data. Nucleic Acids Res. 2019 Jan 8;47(D1):D442-D450.

3. Bruschi M, Granata S, Santucci L, Candiano G, Fabris A, Antonucci N, Petretto A, Bartolucci M, Del Zotto G, Antonini F, Ghiggeri GM, Lupo A, Gambaro G, Zaza G. Proteomic Analysis of Urinary Microvesicles and Exosomes in Medullary Sponge Kidney Disease and Autosomal Dominant Polycystic Kidney Disease. Clin J Am Soc Nephrol. 2019 Jun 7;14(6):834-843.

4. Bruschi M, Granata S, Candiano G, Fabris A, Petretto A, Ghiggeri GM, Gambaro G, Zaza G. Proteomic Analysis of Urinary Extracellular Vesicles Reveals a Role for the Complement System in Medullary Sponge Kidney Disease. Int J Mol Sci. 2019 Nov 5;20(21):5517.
